# An Adrenal Incidentaloma: How Often Is It Detected and What Are the Consequences?

**DOI:** 10.5402/2013/871959

**Published:** 2012-11-28

**Authors:** E. M. Minnaar, K. E. Human, D. Henneman, C. Y. Nio, P. H. Bisschop, E. J. M. Nieveen van Dijkum

**Affiliations:** ^1^Department of Surgery, Academic Medical Center Amsterdam, Meibergdreef 9, G4 1105 AZ Amsterdam, The Netherlands; ^2^Department of Radiology, Academic Medical Center Amsterdam, Meibergdreef 9, 1105 AZ Amsterdam, The Netherlands; ^3^Department of Endocrinology and Metabolism, Academic Medical Center Amsterdam, Meibergdreef 9, F4, 1105 AZ Amsterdam, The Netherlands

## Abstract

*Objectives*. The aim of this study was to investigate the detection rate of adrenal incidentalomas and subsequent workup. *Design*. Retrospective cohort study. *Methods*. Two investigators evaluated the adrenals on abdominal CT scans. Abnormalities were compared to the original radiology reports and an experienced abdominal radiologist reviewed the CT scans. All additional imaging and laboratory tests were assessed. *Results*. The investigators detected 44/356 adrenal incidentalomas (12%). In 25 patients an adrenal incidentaloma had been noted in the radiology report. The expert radiologist agreed on 19 incidentalomas in 17 patients, two with bilateral incidentalomas. Of the 25 incidentaloma patients, 4 (16%) patients were screened for hormonal overproduction and 2 (8%) patients had follow-up imaging studies. *Conclusions*. 12% of the patients had an adrenal incidentaloma (42 of 356). 17 (40%) had initially not been reported by the radiologist. When diagnosed with an adrenal incidentaloma, only a small percentage of patients (16%) is screened or undergoes repeated imaging (8%) as proposed in the National Institutes of Health (NIH) guidelines on adrenal incidentalomas.

## 1. Introduction 

An adrenal incidentaloma is an adrenal mass, larger than 1 cm in diameter, detected on imaging studies performed for other indications than adrenal disease [1, 2]. The increasing use of computed tomography (CT) scans and magnetic resonance imaging (MRI) causes a marked increase in incidence of adrenal incidentalomas [3]. In approximately 6% of all autopsies and 4% of all abdominal CT scans an incidentaloma of the adrenal gland is discovered [4, 5]. The incidence of adrenal incidentaloma increases with age to an incidence of 10% in patients over 70 years old.

Adrenal incidentalomas are characterized by size, growth, imaging characteristics, and functional status. Although rare, the normal function of the adrenal gland can be disrupted by adrenal incidentalomas. In most cases adrenal incidentalomas will be a small, nonhormonal active cortical adenoma, a benign impediment (80%). Some adrenal incidentalomas cause hormonal hypersecretion (15%) or appear to be a primary or secondary malignancy (<5%) [6, 7]. 

Adrenal incidentalomas can cause disease by hypersecretion of hormones. Conditions due to hormonal activity of adrenal incidentaloma include hypercortisolism, (Cushing's syndrome), catecholamine excess (pheochromocytoma), or hyperaldosteronism (Conn's Syndrome) [8].

Subclinical autonomous cortisol hypersecretion is the most frequent hormonal abnormality in patients with adrenal incidentalomas [9]. Some of these patients eventually develop overt clinical Cushing's syndrome. 

Adrenal cortical adenomas with or without hormonal overproduction can only be distinguished by biochemical analysis and not by imaging characteristics. Vascular lesions of the adrenal are suggestive of a medullary derived pheochromocytoma, but confirmation of a pheochromocytoma by serum or urine measurement of metanephrines is required to diagnose pheochromocytoma.

The radiologist plays a central role in the detection of these adrenal incidentalomas. Not only will the radiologist appoint this finding in the radiology report,the radiologist will often also add a recommendation for the next diagnostic procedure.

This retrospective study aims to get a clear view on the detection rate of adrenal incidentalomas on abdominal CT scans in our hospital, and the subsequent diagnostic procedures used after detection of this incidental finding.

## 2. Methods

This retrospective study was designed to investigate the detection rate of adrenal incidentalomas on abdominal CT scans. For inclusion in this investigation, an adrenal incidentaloma was defined as an adrenal mass, greater than 1 cm in diameter, initially discovered by diagnostic imaging for a clinical condition not related to, or suspicious for, adrenal disease. Two investigators learned from an expert radiologist to examine CT scans of the abdomen for primarily the adrenal gland. Each investigator independently evaluated 180 CT scans out of 360 CT scans, of many patients, that were indicated for diagnostics of hepatic, pancreatic, or renal pathology between 2005 and 2007. CT scans indicated for adrenal disease were excluded. Age, gender, indication for CT scan, and abnormalities in size or morphology, were noted. 

At the time of examining the adrenal glands, the investigators were not aware of the content of CT scans' radiology report or indication for CT scan. Only after their own judgement about size and aspect of the adrenal glands, indication for CT scan and the radiology report were read and noted. 

An expert abdominal radiologist (over 45,000 abdominal CT scans examined) reviewed the CT scans marked by the investigators with abnormal adrenal glands in size or morphology and results were compared. If an adrenal incidentaloma was mentioned in the original radiology report, the patients' record was reviewed to determine whether additional investigations, for example, hormonal studies, additional imaging, or interventional diagnostic studies, were performed after the CT. 

An interobserver test was performed to evaluate the resemblance of the way of evaluation between the investigators and expert radiologist. The CT scans performed for suspected hepatic, pancreatic, or renal pathology of 75 new patients, between 2007 and 2008, were evaluated for this purpose.

The interobserver variability was calculated with the Friedman test, a non-parametric test for coupled observations, with independent observers. Statistical significance was determined using *t* tests and Chi-square analysis. *P* < .05 was considered significant.

## 3. Results

Of the 360 patients studied, 206 (57%) were men and 154 (43%) were women. The age ranged from 21 to 88 years with an average of 55 years. Of the total of 360 patients, 4 patients were excluded because suspected adrenal pathology was also an indication for imaging in these patients.

In the remaining 356 CT scans evaluated, the investigators discovered independently a total of 44 (12%) abnormal adrenal glands in 42 patients (2 bilateral adrenal incidentalomas). Each radiology report was checked for adrenal incidentalomas which were already noted. In 25 (7%) of 356 patients an adrenal mass was already noted in the radiology report.

**Figure 1:**
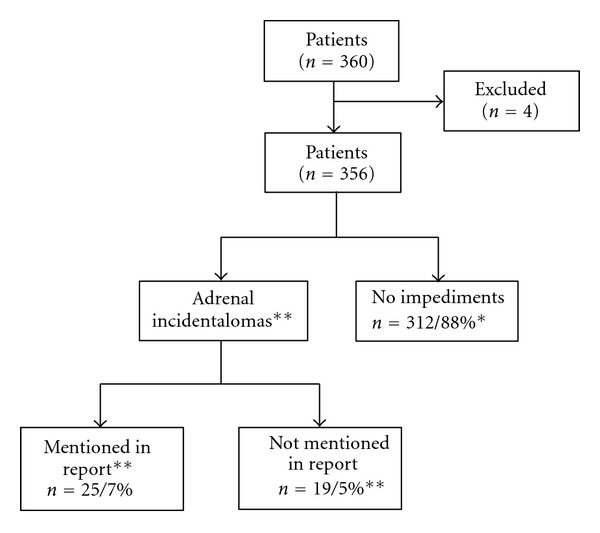
Results of the study.  * Investigated  by researchers, ** confirmed  by  radiologist.

The expert radiologist reassessed the CT scans of 42 patients; the radiologist discovered 17 patients with an adrenal incidentaloma. Two patients had bilateral incidentalomas, giving a total of 19 adrenal incidentalomas not noted previously (Figure 1). The total number of adrenal incidentalomas was 44/356 (12%) in 42 patients. 

Figure 3 shows an enlarged right adrenal not mentioned in the initial radiology report. In 64 of 356 (18%) patients, a malignancy was the indication for imaging. Patients with an adrenal incidentaloma were more likely to have a malignancy as indication for CT scan, 20/42 (48%). 

The 25 patients that had an adrenal incidentaloma mentioned in the initial radiology report were checked for additional diagnostic procedures performed. In 3/25 patients a follow-up CT scan was recommended, this CT was performed in 2 patients.

A total of 4 (16%) patients were referred to the endocrinologist. None of these patients showed hormonal overproduction caused by the adrenal lesion. In 2 patients (8%) a second CT scan was performed to exclude increase in adrenal size; there was no growth of the adrenal lesion shown. 

One patient who underwent a hemihepatectomy had a simultaneous resection of the right adrenal incidentaloma. Pathological examination of this lesion showed an adenoma. This patient was not screened preoperatively for hormonal overproduction.

### 3.1. Interobserver Variability

The interobserver variation was calculated using a Friedman analysis test to exclude or to demonstrate a significant difference in perception between different readers. The Friedman test showed that there was no statistically significant difference in perception between the investigators and the radiologist (*P* = .867).

## 4. Discussion

The detection of adrenal incidentalomas in this study was 7% and the actual incidence of adrenal abnormalities was 12% after focused assessment of CT scans. This is high compared to other studies and can be explained by the relatively large group of patients who underwent imaging examination because of a malignancy [7, 11]. However, because of the absence of histological examination of the enlarged adrenal glands, it cannot simply be concluded that these patients had metastases in the adrenal gland. The incidence of adrenal incidentalomas in the literature varies from 0.5% to 15% and depends mainly on the age of the investigated group [11, 12].

The adrenal incidentaloma is a common incidental finding in our hospital. However, there is no uniform policy for subsequent diagnostic procedure in these patients. Only 6 of 25 (24%) patients with an adrenal incidentaloma were further investigated with hormonal workup or imaging.

One reason for the lack of additional diagnostic testing and treatment after detection of an adrenal incidentaloma is the lack of a clear evidence based guideline. Also unfamiliarity with adrenal incidentalomas by the physician who has requested the CT scan will cause lack of additional screening. This incidental finding complicates the diagnostic process of patients and causes delays and uncertainty.

Another reason for the lack of additional diagnostic testing of adrenal incidentalomas is the apparent lack of direct clinical consequences [13, 14]. Undetected hormonal hypersecretion will probably reveal itself in time and the chance of an adrenal carcinoma is low with 0.72/million/year [10, 13].

Because of this low risk of malignancy and because of the limited impact of hormonal overproduction in an asymptomatic patient, it is not clear whether a guideline for diagnosis and treatment of adrenal incidentaloma is necessary. Such a guideline may cause an increase in diagnostic procedures with additional burden and uncertainty for the patient. A comprehensive cost-effectiveness study showed however that hormonal analysis of an adrenal incidentaloma is cost effective [14]. The cost effectiveness of additional imaging of the adrenal gland is less clear in this study. In addition, the radiation dose to the patient becomes more important and therefore complementary imaging with an MRI scan has its advantage [15].

The optimal strategy for screening and followup of an adrenal incidentaloma is still under discussion. The guideline of the NIH (2002) seems to be the best alternative in this debate, but the NIH already recognized that the guideline is not based on hard evidence [10]. (Table 1) Protocols on the diagnostic procedures of adrenal incidentalomas are described in several other publications as well, without describing additional prospective data on the clinical results of these protocols [9, 10, 15–18]. There are currently no prospective studies that have examined the effects of an additional diagnostic procedure for adrenal incidentalomas. 

**Figure 2:**
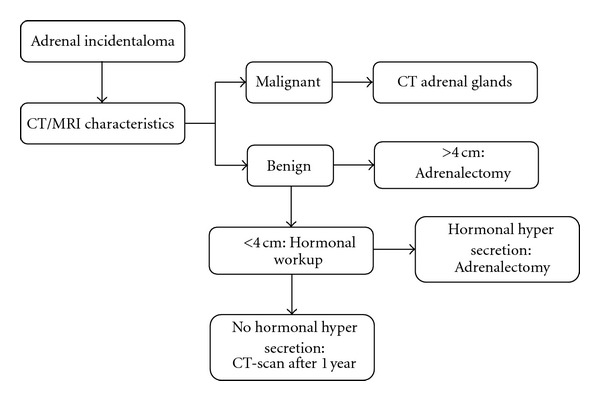
Suggested flowchart for evaluation of an incidentally discovered adrenal incidentaloma.

**Figure 3:**
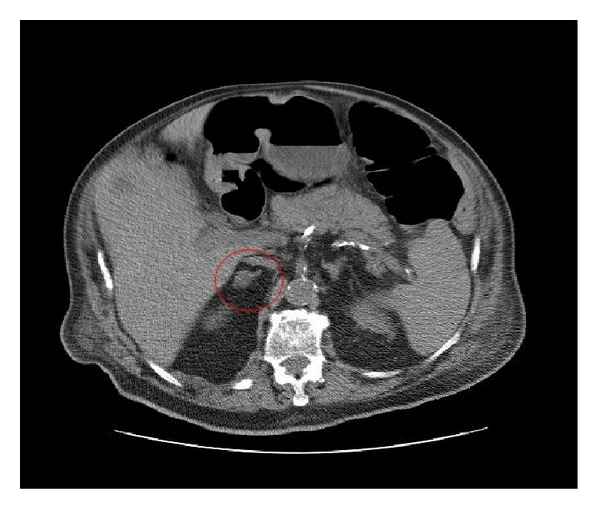


Following this study a guideline for additional screening and followup was made (Figure 2). In each radiology report a recommendation will be added for every patient with an adrenal incidentaloma and the referring physician is pointed to a webpage with a brief summary about adrenal incidentalomas. This website also contains the brochure with information for patients, which can be printed. 

In conclusion, an adrenal incidentaloma is a relatively common finding on abdominal CT. It is not always noted by the radiologist and focused assessment of abdominal CTs increased the detection rate of this abnormality form 7% to 12%. When detected and mentioned in the radiologist's report, only a small percentage of patients receives additional hormonal or imaging investigations to determine the nature of the incidentaloma.

## Figures and Tables

**Table 1:** NIH guideline [10].

	NIH 2002
Functional adrenal incidentaloma (hormonal hypersecretion)	Surgical resection or medical treatment
Nonfunctional adrenal incidentaloma	>6 cm: surgical resection
	4–6 cm: resection or followup
Nonfunctional adrenal incidentaloma < 4 cm	No surgical resection, no recommendations about followup
Adrenal incidentaloma suspicious for metastasis	No benefit from resection
